# Decriminalization in action: lessons from the Los Angeles model

**DOI:** 10.1017/S1092852919001561

**Published:** 2020-10

**Authors:** Kristen Ochoa, Oona Appel, Viet Nguyen, Elizabeth Kim

**Affiliations:** 1 Office of Diversion and Reentry, Los Angeles County Department of Health Services, Los Angeles, California, USA; 2 Department of Psychiatry and Biobehavioral Sciences, David Geffen School of Medicine at UCLA, Los Angeles, California, USA

**Keywords:** Diversion, Mental health, Jail, Office of Diversion and Reentry, Decriminalization

## Abstract

Los Angeles County’s Office of Diversion and Reentry (ODR) has removed over 3800 people from the largest jail system in the country. Across various diversion programs, ODR’s fundamental goal is to provide permanent, lifetime care for each diverted person. This article describes ODR’s various diversion programs, and elucidates the types of elaborate clinical and court-related interventions that are necessary to remove persons with serious mental disorders from jail custody. As Los Angeles continues to build the necessary community-based continuum of mental health care, ODR’s model proves that thoughtfully removing persons with serious mental disorders from jail is possible and necessary for the health of both patients and community.

*“The insane criminal has nowhere any home: no age or nation has provided a place for him. He is everywhere unwelcome and objectionable. The prisons thrust him out; the hospitals are unwilling to receive him. And yet humanity and justice, the sense of common danger, and a tender regard for a deeply degraded brother-man, all agree that something should be done for him—that some plan must be devised different from, and better than any that has yet been tried, by which he may be properly cared for, by which his malady may be healed, and his criminal propensity overcome.”*

Jarvis E. Criminal insane: Insane transgressors and insane convicts. *Am J Psychiatry*. 1857;13:195–231.

## Introduction

A home and treatment. And not giving up on anybody. That is what we do in Los Angeles to decriminalize mental illness. Our model stands on philosophies of harm reduction and housing first. It is an admittedly basic approach, but has worked to remove thousands of persons with serious mental disorders from our vast jail system. This approach asserts that many persons are in jail because of a systematic failure to adequately care for them. It is an approach that re-creates systems to provide permanent care, without rationing, for the lifetime of the patient. To accomplish this, the new system must say “Yes” with the same veracity and availability of a jail bed, and with the same ease and routine of a jail booking. It has to be flexible and forgiving; it has to be a commitment to “care first, jail last.”

This is not to say that we are not realists. We understand that not everyone can avoid jail, for some have crimes too serious. We also understand that, if sufficient community services existed, more than half of the jail mental health population could be released. On any given day, Los Angeles jails over 16,000 people. More than 5000 of those reside in the mental health section of the jail. Providing an alternative place for those with serious mental disorders to live and thrive is a tremendous undertaking. Los Angeles has only begun to build a continuum of care to accommodate this need, but the concept proves that thoughtfully removing persons with serious mental disorders from the jail is possible and necessary for the health of both patients and community.

In addressing one of the greatest public health problems of our time, we can learn from other scientists. Consider ecologists, who are concerned with interrelationships and their impact on the environment. When ecologists create reserves, they reconnect habitats that have been fragmented, allowing them to function as a whole rather than as a set of independent pieces. The Los Angeles model works to create that “reserve” through the Office of Diversion and Reentry (ODR), which connects institutions and justice partners on clinical matters and public health solutions. Through elaborate clinical interventions on behalf of persons with serious mental disorders in custody, connections are made between jail and court, community and hospital, clinic and housing, and back again.

## ODR’s Community-Based Programs

**Figure 1.**
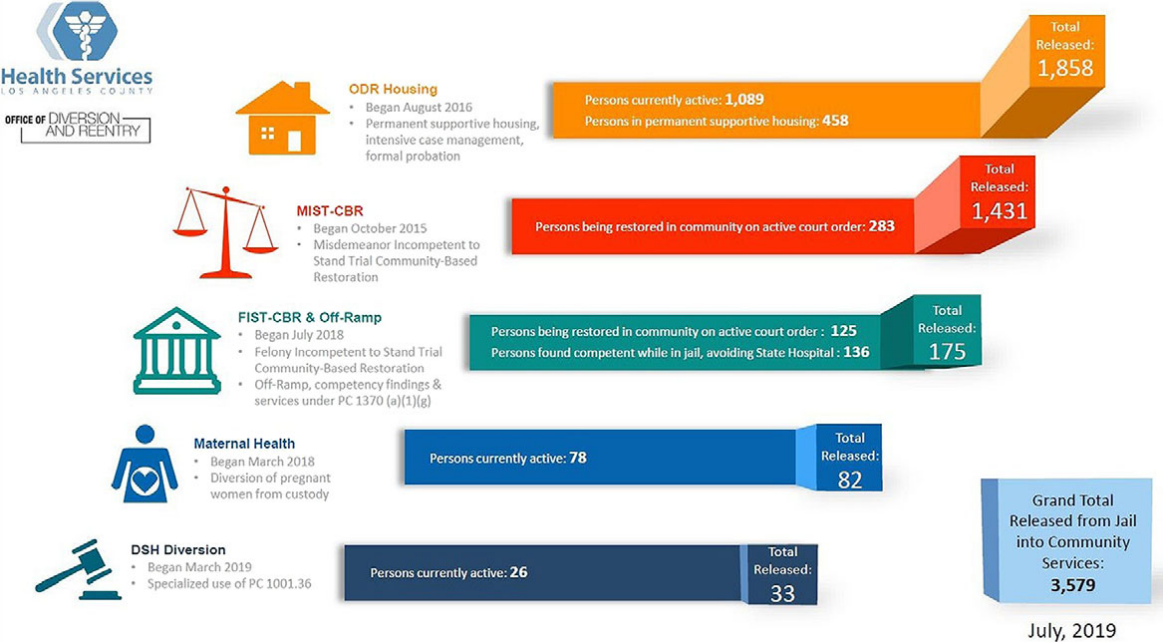
Office of Diversion and Reentry diversion dashboard.

At the time of this writing, ODR has diverted more than 3800 persons with serious mental disorders from the Los Angeles County Jail system. Within ODR, diversion is conceptualized as circumventing or significantly reducing jail time through linkage to housing and community treatment services. All ODR programs consist of 3 primary interventions: jail in-reach services, enhanced treatment efforts provided by ODR clinical staff (additional clinical assessments and initiation of medications, as indicated), and immediate interim housing upon release in anticipation of permanent supportive housing (see Figure 1).

**FIGURE 2.**
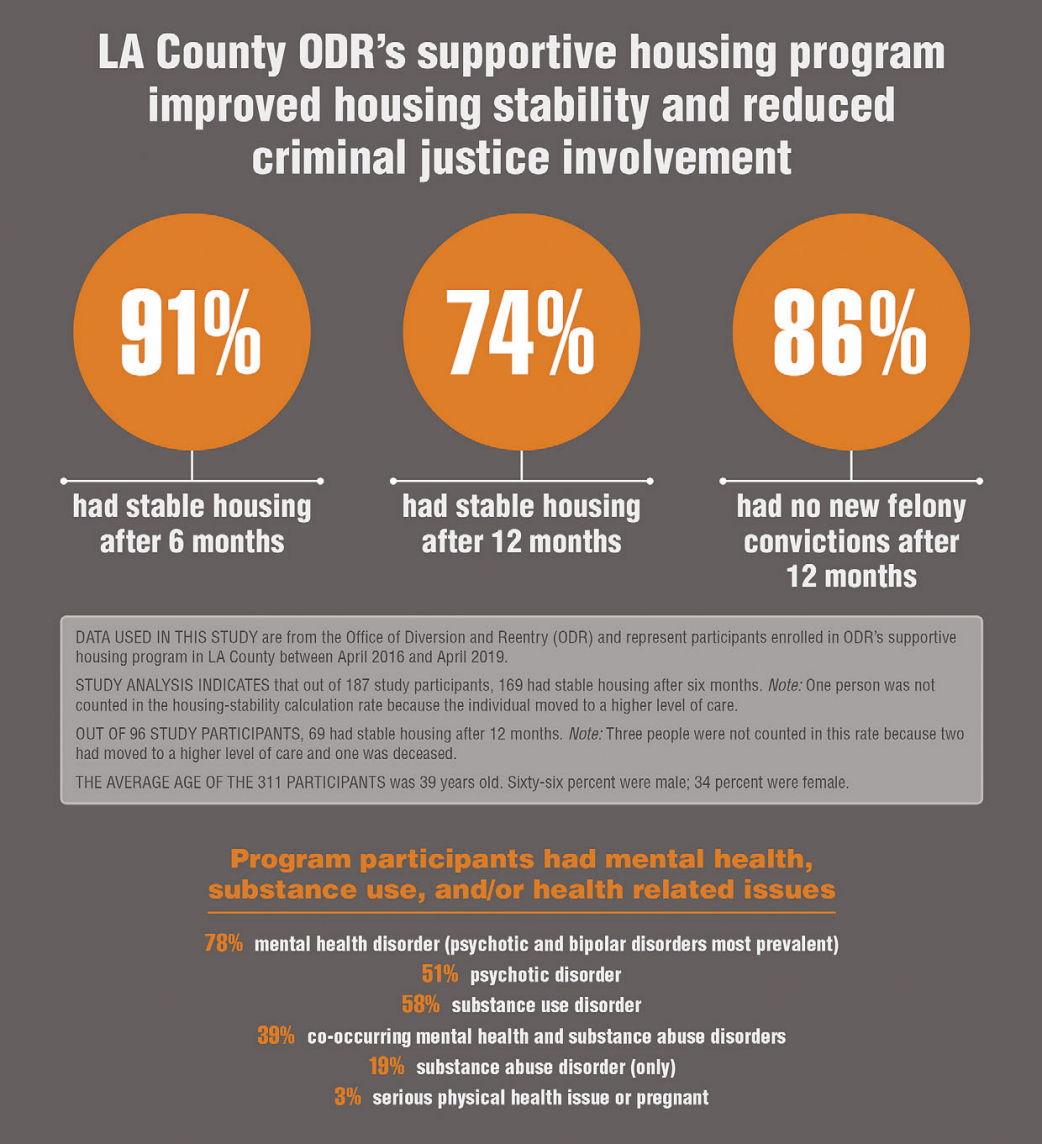
Preliminary data on ODR’s permanent supportive housing program.^6^

The ODR Housing program provides permanent supportive housing to individuals who are homeless, have a serious mental health disorder, and who are incarcerated in the Los Angeles County Jail. This program, offered in partnership with the Los Angeles County Superior Court, is available to pretrial defendants who have criminal felony cases. ODR Housing attempts to resolve these felony cases early and divert defendants through a grant of probation. Clients in the ODR Housing program are assigned an Intensive Case Management Services (ICMS) provider who works with the client as they transition from custody to community. ICMS providers serve as the core point of contact for supportive services including medical and mental health treatment. See Figure 2 for preliminary data on permanent supportive housing.

There are 2 programs that divert individuals found incompetent to stand trial; one program for those charged with misdemeanors, and one for those charged with felonies. These defendants are diverted into community-based settings for competency restoration treatment. Settings are tailored to meet the needs and clinical acuity of the clients; placements range from acute inpatient to open residential settings.

California Assembly Bill 1810 and Senate Bill 215 amended the California Penal Code (PC 1001.36) to create a pathway for courts to authorize pretrial diversion. ODR’s specialized use of PC 1001.36 is funded by the Department of State Hospitals (DSH), and diverts clients who are charged with felonies, have a serious mental disorder, and who may be found incompetent to stand trial if there were no clinical intervention. This program, called DSH Diversion, provides supportive housing, intensive case management, medical and mental health treatment, and specialized pretrial probation services. Upon completion of this program, the charge(s) will be dismissed.

Under the directive of the Los Angeles County Board of Supervisors, ODR created the Maternal Health Program. This program diverts pregnant women from jail to community through providing housing and supportive services. The majority of pregnant women served by ODR reside in specialized interim housing settings that allow women to remain with their children until they are moved to permanent supportive housing.

Jail diversion of people with serious mental disorders requires a continuum of care in the community. Currently, ODR’s highest level of care is a dedicated psychiatric inpatient unit at Olive View-UCLA Medical Center (OVMC). Patients in this 18-bed unit are transported directly from the jail through ODR’s clinical and court interventions. Prior to transport, they are among those with the highest symptom acuity in the jail, and are typically waiting for admission to the jail’s psychiatric inpatient unit. Once a patient is transferred from the jail and stabilized at OVMC, they are moved to one of ODR’s community-based programs.

Complex clinical and court interventions are required to divert people into the aforementioned programs. ODR’s team works across spaces and disciplines to support the adversarial legal process, provide direct clinical care, and conduct forensic evaluations to hasten and expand diversion efforts.

## Adversarial Process

ODR’s clients have serious mental disorders, are homeless but for their jail bed, and are involved in pretrial legal proceedings. A client’s admission to an ODR program is done through court order, thus legal components are intrinsic to the work. The ODR Housing program currently operates out of 2 different courts (“Hubs”), which receive cases from courthouses in nearby jurisdictions (“Feeder Courts”). After a defense attorney refers a client, ODR conducts a brief review of the case and ensures it is in the correct legal posture to proceed. Through reviewing both jail and community medical records, ODR’s clinical team then completes a comprehensive assessment to identify the presence of a serious mental disorder and/or serious medical need. When it remains unclear whether the client meets clinical eligibility criteria, ODR staff meets with the client in jail and/or gathers information from additional collateral sources. This understanding of the client’s clinical needs informs ODR’s legal interventions. If a client is deemed initially eligible clinically, ODR submits a recommendation to the court and a hearing for legal suitability is calendared. Legal suitability hearings dictate the actual disposition of a case and are by definition adversarial. However, the parties have a common goal of diverting clients with serious mental disorders from jail into appropriate treatment. When the prosecutor and defense attorney do not agree on diversion, or the judge requests a better understanding of the client, ODR serves as a neutral advisor to all parties in the courtroom. As neutral advisor, ODR helps legal partners transcend the adversarial proceedings to inform a truly collaborative effort.

## Clinical Courtrooms

The role of the ODR psychiatrist is unique. They are differentiated from the correctional health psychiatrist, who often has no stake in the patient’s release into the community, and additionally differentiated from the traditional forensic expert, who has no involvement in treatment. The ODR psychiatrist evaluates and manages treatment by first envisioning the patient’s circumstances out of custody, then ensuring they are stable for community treatment, and finally liaising and consulting with community partners to ensure stability after release. To effectively identify, evaluate, and manage patients with serious mental disorders, the ODR psychiatrist must collaborate with justice partners, the Sheriff’s department, correctional health services, and Los Angeles County’s public mental health system.

Patients cannot fully engage with community services if their symptoms, often exacerbated within the jail, are not treated and stabilized prior to release. The ODR psychiatrist plays an important role in treating and stabilizing identified clients through providing evidenced-based, high-quality treatment within the correctional setting prior to release. Given that treatment adherence is a common issue when patients are in the community, the use of long acting injections (LAIs) is prudent. In large cohort studies, LAIs have been shown to reduce mortality in patients with schizophrenia^1^ in addition to reducing relapse in symptoms compared with oral agents.^2^ Along with permanent supportive housing, LAIs are a crucial part of a typical ODR client’s treatment plan.

For the ODR Housing program, ODR psychiatrists are present in specialized courts that serve the entirety of Los Angeles County’s criminal court system. Once candidates have gone through the initial legal and clinical screening, they present to court. In the court’s lock-up area, ODR psychiatrists evaluate patients who are potentially unstable in the jail, have questionable medication regimens, are not prescribed medications at all, or whose difficulty following the rules and conditions of probation could be addressed by a medication evaluation (eg, oral medication non-compliance in the community). ODR psychiatrists have remote access to the jail electronic medical record, and can place orders in real time after a client’s case has been resolved.

Moving clinical care and treatment to the courtroom allows for enhanced collaboration between justice partners, ODR, and the patient. In addition to evaluating and potentially treating candidates, the ODR psychiatrist provides clinical expertise to the court in helping create individualized treatment plans inclusive of housing, linkage to outpatient mental health treatment, and other rehabilitative services. Likewise, probation terms can be tailored to individual needs if there is clinical expertise advising the court. This collaborative process is critical in helping ODR successfully divert patients with serious clinical needs into the community.

## Forensic Evaluations

As highlighted above, ODR’s work is at the intersection of mental health and the law. In addition to providing court consultation and direct clinical services, ODR staff conducts both traditional forensic evaluations (eg, competency to stand trial) and quasi-forensic evaluations (eg, jail diversion evaluations) to assist in diversion efforts. ODR’s evaluators, including a forensically trained psychiatrist and psychologist, maintain core principles of forensic evaluation including objectivity and awareness of relevant law and ethics. Simultaneously, evaluations align with ODR’s fundamental mission to reduce the population of those with serious mental disorders in the jails. ODR uses forensic evaluations as one tool in recreating the public mental health system, allowing practitioners to think more broadly about how their work impacts individuals and communities.

In California, as in many states, there has been a marked increase in defendants found incompetent to stand trial. It is well established that defendants found incompetent to stand trial languish in local jails while awaiting appropriate treatment.^3^ ^–^ ^5^ Those found incompetent on felony charges in California join a lengthy waitlist for admission to DSH. To address this waitlist, ODR and DSH created new pathways for felony defendants found incompetent to stand trial. The Felony Incompetent to Stand Trial Community Based Restoration (FIST-CBR) program was created in an effort to divert felony defendants with serious mental disorders out of the jail and into community care while under court supervision. FIST-CBR provides housing, competency restoration treatment, case management, individual and group therapy, and medication management. This small-scale deinstitutionalization effort treats defendants in the community rather than in the penal or state hospital system.

Additionally, in 2018 ODR worked with legal partners to amend the California Penal Code. The amendment, PC 1370(a)(1)(g), states that if the symptoms of a defendant found incompetent to trial “have changed to such a degree as to create a doubt in the mind of the judge as to the defendant’s current mental incompetence, the court may appoint a psychiatrist or a licensed psychologist to opine as to whether the defendant has regained competence.” Subsequent to this amendment, ODR staff evaluates incompetent felony defendants who may have regained competency while waiting for DSH placement. If defendants are competent, these evaluations help move their cases forward while also decreasing the burden on DSH. Moreover, by not sending competent defendants to DSH for restoration, more state hospital beds are open for patients who truly need that higher level of care.

ODR also submits competency evaluations for the most psychiatrically acute defendants in jail. These defendants often miss court due to symptom acuity, and are left with their cases being continued, resulting in prolonged detention for those with serious mental disorders. ODR tracks the legal proceedings of the most psychiatrically acute defendants in jail, and intervenes by evaluating these defendants in jail if they miss their competency-related court dates. Through these evaluations, ODR assists in hastening psychiatric treatment while also moving criminal proceedings forward. Moreover, those defendants whom ODR opines incompetent to stand trial become eligible for transfer to ODR’s psychiatric inpatient unit at OVMC. Once stabilized on the inpatient unit, the defendants are transitioned to community-based restoration in an open residential setting.

## Conclusion

It will take years of work to stem the tide of mass incarceration and create the necessary community resources to divert those who are homeless with serious mental disorders. Recognizing this staggering challenge, Los Angeles now has a large-scale and effective alternative to incarceration through the work of ODR. A growing commitment to scale diversion may radically decriminalize and deinstitutionalize the largest jailed population in the United States.
